# Abdominal Injuries from Civilian Conflicts: An Emerging Global Health Challenge in Rural Southeast Nigeria

**DOI:** 10.5334/aogh.3973

**Published:** 2023-01-27

**Authors:** Aloysius Ugwu-Olisa Ogbuanya, Nonyelum Benedett Ugwu, Nwanneka Kwento, Edwin Ifeanyi Enyanwuma, Ferdinand Friday Anyigor, Utom Oko

**Affiliations:** 1 Department of surgery, Alex Ekwueme Federal University Teaching Hospital, Abakaliki (AEFUTHA), Ebonyi State, Nigeria; 2 Department of Surgery, Ebonyi State University, Abakaliki (EBSU), Ebonyi State, Nigeria; 3 Department of surgery, Bishop Shanahan Specialist Hospital, Nsukka, Enugu state, Nigeria; 4 Department of Surgery, Mater Misericordie Hospital, Afikpo, Ebonyi State, Nigeria; 5 Department of Surgery, District Hospital, Nsukka, Enugu State, Nigeria; 6 Department of Nursing Sciences, University of Nigeria Teaching Hospital, Enugu, Nigeria; 7 Department of surgery, Mater Misericordea Hospital, Afikpo, Ebonyi state, Nigeria

**Keywords:** Abdominal wounds, armed robbery, civilian violence, gunshot, mortality

## Abstract

**Background:**

In recent time, incidence of abdominal injuries continues to increase steadily in most major regions of West Africa due to emergence of various religious, social and political conflicts. Indeed, violence and social conflicts constitute major global public health challenges that commonly lead to injuries and long-term physical and mental health problems. In our setting, increasing cases of abdominal trauma resulting from civilian violence led to additional workload in the general surgery unit and the audit of our experiences is presented in this paper.

**Objective:**

To analyze the etiological spectrum, trend and management outcome of abdominal injuries from civilian violence in our setting.

**Methods:**

This was a multicenter prospective study of adult patients with abdominal injuries from civilian conflicts managed at three selected district hospitals in Southeast Nigeria between January 2013 to December 2020.

**Findings:**

Of 398 patients evaluated, 359 (90.2%) sustained penetrating while 39 (9.8%) had blunt abdominal injuries. Gunshot was the most common mechanism, accounting for 248 (62.3%) cases, followed by stab wound (95, 23.9%). Armed robbery attack (68, 27.4%) was the main source of gunshot wounds. Overall, annual rates showed a four-fold rise over an eight-year period from 24 cases (6.0%) in 2013 to 96 (24.1%) in 2020. Majority (365, 91.7%) had operative management (OM); the rest (33, 8.3%) were treated non-operatively. Morbidity and mortality rates for operative cases were 29.6% and 12.1% respectively. The main factors associated with increased mortality rates were delayed presentation (p = 0.002), bowel resection (p = 0.006), gunshot wounds (p = 0.013), advanced age (p = 0.033), multiple visceral injury (p = 0.034) and ASA score ≥ III (p = 0.001).

**Conclusion:**

Abdominal trauma from civilian violence is on the steady rise in our setting. The main etiologic factors are armed-robbery, communal clashes, political thuggery and cultism perpetrated predominantly through gunshots and stab wounds. Advancing age, gunshot wounds, delayed presentation, bowel resection and multiple injuries were associated with increased mortality.

## Introduction

The incidence of abdominal trauma continues to rise steadily in most major cities in West Africa due to emergence of various predisposing social and political factors, leading to civilian unrest and injuries [1,2,3,4]. Briefly, trauma is a major cause of morbidity and mortality worldwide, representing 16% of global disease burden and 10% of mortality; the injuries occur most commonly in those aged 15–45 years [2,5,6]. Curiously, over 90% of injuries occur in low and middle-income countries (LMICs) and Africa, mainly sub-Saharan region, contributes 21% of this quota [5,6]. Despite these disturbing rates and overbearing unmet needs for most surgical conditions, only 6% of 313 million procedures undertaken worldwide each year occur in the poorest countries where over a third of the world’s population lives [3]. The World Health Organization (WHO) global burden of injury estimate ranks injury among the top ten leading causes of death, with an estimated five million deaths annually of which men in Africa have the highest injury-related mortality rates in the world [3,7,8,9,10].

Abdominal trauma from civilian violence is not an African phenomenon, but occurs all over the world, though the etiological factors may vary from place to place and between peace time (civilian conflicts) and war time [3,5]. In the past, gunshot injuries in our setting were rare before the Nigerian civil war (1967–1970), but armed-robbery and other crime-related gunshot injuries became very rampant in recent years in the urban, semi-urban and rural areas of the country [1,3,4,11]. In contrast, gunshot injuries (GSIs) in developed nations were largely due to homicidal and suicidal fire-arm related violence [10,12]. Indeed, recent published data indicate that religious violence is the common cause of GSIs in Northern Nigeria, while armed robbery-related GSIs are common in Southern Nigeria [1,2,3,11]. Additionally, GSIs in Nigeria may result from terrorist activities, communal clashes, cultism, political thuggery, assassination and accidental shots fired by law enforcement agents and during celebrations, festivals or hunting expeditions [3,11,13,14,15]. Unfortunately, abdomen is particularly vulnerable to both blunt and penetrating injuries from civilian conflicts [1,8,14]. It is estimated that approximately one-third of all trauma patients have abdominal injuries though a careful triage will often isolate approximately 25% of such injuries for exploratory laparotomy [16,17].

In recent time, many abdominal injuries especially those involving solid organs are managed non-operatively in centres where imaging facilities like ultrasonography, computerized tomography (CT) scan and magnetic resonance imaging (MRI) are available and operational [1,5,16,17,18]. Regrettably, CT scan and MRI facilities are either lacking or if available, not operational in many private and government owned hospitals in sub-Saharan Africa and other developing economies, thereby making conservative management (requiring serial clinical and radiological assessments of injured organs) a major challenge [5,16,19]. Nevertheless, surgeons in the resource-poor regions of the world face these additional challenges and often time will have to grapple with the dilemma of whether to “operate” or “not to operate”. Under this scenario, facilitated consultation, led by a dedicated general surgical team is expedient and rewarding.

In a rural set up where patients are poor and more likely to present late, a three-pronged problem of limited health facilities, low workforce and deplorable road networks makes management of the patients more difficult. More recently, increased numbers of surgical admissions from civilian conflicts were observed in the selected district hospitals in Southeast Nigeria where this study was carried out. Moreover, no studies on abdominal trauma due to civilian conflicts at district setting in Southeast (SE) Nigeria were reported previously. Existing data are non-specific to abdomen and skirts through subjects like missiles and blasts, gunshots and orthopedic injuries [3,10,19]. The aim of this study is to determine causes, trend and outcome of management of abdominal injuries due to civilian violence at district centers in Southeast Nigeria.

## Methods

### Design and setting

This is a prospective observational study involving all consecutive adult patients with abdominal injuries from civilian unrest recruited over eight years period from January 2013 to December 2020. All the patients were managed at the three hospitals in southeast Nigeria.

### Subjects

All adult patients aged 16 years and above who gave consent were included. Selection criteria included abdominal injuries strictly from civilian violence which occurred during armed robbery attack, banditry, insurgency, political and religious conflicts and other forms of civilian unrest. Abdominal injuries arising from road traffic accidents (RTA), industrial accidents, recreational injuries and unintentional wounds from sharp objects, gunshots and fall from heights were excluded. Those who withheld detailed clinical history or who died before initial assessment were also excluded.

### Procedure

All recruited patients were initially resuscitated. Their sociodemographic and clinical data were recorded in a proforma. Detailed history on the mechanism of injuries, delay before presentation, specific causes of injuries (armed robbery, communal clashes, cult activities, farmer-herder clashes), comorbidities and associated extra-abdominal injuries were recorded. Delayed presentation was estimated from time of injury until presentation at the hospital. At the time of admission, history of pre-hospital care and hemodynamic parameters were noted. All recruited patients did basic hematological tests, serum electrolytes and urinalysis. Those scheduled for non-operative management (NOM) were required to do serial abdominal ultrasound scans and clinical assessment. Computed tomography (CT) scan were done by only few of those managed by NOM due to high cost, unavailability and non-functional status of few available centres.

Preoperatively, patients selected for operative management (OM) were subjected to thorough anaesthetic assessment and their injuries categorized (blunt abdominal trauma–BAT, or penetrating abdominal trauma-PAT) accordingly. Intra-operatively, the organ(s) involved and the operative techniques performed were noted and entered into a proforma. Early post-operative complications and length of hospital stay (LOHS) were recorded. Patients were followed up for a variable period of three to 36 months. During follow up, late postoperative complications were recorded. All follow up data including complications were recorded in a proforma. Patients who failed to keep follow up appointments for two consecutive times were routinely contacted directly or through relatives via telephone interviews or text messages. All deaths were recorded.

### Data analysis

Data analysis was done using Statistical Package for Social Science (SPSS) Software version 22.0 (IBM, CHICAGO, IL, USA 2015). For the categorical variables, data were summarized in proportions and frequency tables. For continuous variables, we computed the ranges and mean. During analysis, we computed p-values for categorical variables using Chi-square and Fisher’s exact test in accordance with the size of the dataset. We also determined the association between some selected clinical variables and mortality or morbidity using logistic regression analyses. Confidence interval was calculated at 95% level and significance at 5% probability level (P < 0.05).

### Ethical Approval

The protocol for this study adhered to the “Declaration of Helsinki” and was approved by the “Ethical Research Board” of the hospitals. The reference number for Bishop Shanahan Hospital is BSH/ERB/12/24. The reference numbers for Mater Misericordia Hospital and District Hospital Nsukka are MMH/ADM/11/14 and DHN/ENU/RB/12/04 respectively.

## Results

### Sociodemographic characteristics

During the period of study, a total of 769 patients with abdominal injuries were seen, but only 446 (58.0%) were due to civilian violence; the rest (323, 42.0%) were from RTA, accidental fall from heights, industrial accidents, domestic accidents and recreational injuries. Of the 446 cases, 48 (10.8%) patients were excluded from further evaluation. These included 12 that died before detailed clinical assessment, 31 with anterior abdominal wall lacerations and other minor injuries not considered for admissions and 5that failed to give consent. The remaining 398 (89.2%) patients formed our study population and were further assessed. The ages of the patients ranged between 16 to 72 years with a mean of 32.4 ± SD 16.48. There were 312 (78.4%) males and 86 (21.6%) females giving male to female ratio of 3.6:1. The vast majority (309, 77.6%) were aged 50 years and below. Over three-quarters (304, 76.4%) were rural dwellers. Majority (221, 55.5%) were farmers; the rest were traders (78, 19.6%), artisans (57, 14.3%) and others (42, 10.6%).

### Mechanism of injury and clinical presentations

Majority (359, 90.2%) arose from PAT while 39 (9.8%) were due to BAT. Gunshot wounds (248, 62.3%) were the most common injury mechanism, followed by stab wounds (95, 23.9%), then assaults/domestic violence (41, 10.3%), machete cut (7, 1.8%), push from height (5, 1.3%) and injuries sustained during rape (2, 0.5%). Of the 248 cases from gunshot injuries, armed robbery attack (68, 27.4%) was the main source. The details of the mechanisms of injuries and their specific causes are shown below (Table 1). The rates of the injuries showed an upward trend from 2013 to 2020 (Table 2). Majority (189, 47.5%) of the patients presented after 24 hours of injuries. Only 97 (24.4%), patients presented within six hours of injuries; the remaining 112 (28.1%) were seen between 7–24 hours after injury. About one-fifth (78, 19.6%) had associated extra-abdominal injuries. The order of frequency of barriers to early presentation in the 301 (75.6%) patients that presented after 6 hours of injury include delayed referral by private hospitals (102, 33.9%), treatment by non-physician clinicians (78, 25.9%), herbalists (49, 16.3%) or patent medicine dealers (32, 10.6), failure of patients and relatives to appreciate the clinical magnitude of the abdominal injury (28,9.3%) and missed injuries due to poor assessment (12, 4.0%). At the time of initial presentation, nearly two-third (260, 65.3%) of the entire patients were in shock with systolic blood pressure ranging from 40–80 mmHg and pulse rates of 90–14 beats/min. Of these, 257 (98.5%) were managed by OM; all the 257 patients received blood transfusion both preoperatively and intraoperatively. The remaining three patients who presented in shock, but managed non-operatively also received blood transfusion to reverse the hemodynamic instability. Of the 138 (34.7%) that were hemodynamically stable at presentation, 108 (78.3%) received OM and were all transfused intraoperatively. Put differently, 30 of the 33 patients managed by NOM were not transfused; all (365, 91.7%) patients managed operatively received blood transfusion in the preoperative, intraoperative or postoperative period.

**Table 1:** Specific causes of abdominal injuries.


SPECIFIC ETIOLOGIC CAUSE	MECHANISM OF INJURIES				

	GUNSHOT	STAB	ASSAULT	OTHERS	TOTAL (%)

Armed Robbery	68	21	2	0	91 (22.9)

Communal clashes	39	21	4	2	66 (16.6)

Political thuggery	30	14	8	2	54 (13.6)

Cult activities	24	15	6	2	47 (11.8)

Farmer-Herder clashes	28	13	1	1	43 (10.8)

Kidnapping/Banditry	39	2	0	0	41 (10.3)

Law enforcement Agency	20	0	0	0	20 (6.0)

Domestic violence	0	4	16	2	22 (5.0)

Public fights	0	5	4	3	12 (3.0)

Rape	0	0	0	2	2 (0.5)

Total	248 (62.3)	95 (23.9)	41 (10.3)	14 (3.5)	398 (100.0)


**Table 2:** Annual trend of abdominal injuries.


YEAR	MECHANISM OF INJURIES				

	GUNSHOT	STAB	ASSAULT	OTHERS	TOTAL (%)

2013	12	7	4	1	24 (6.0)

2014	13	7	3	2	25 (6.3)

2015	15	8	4	1	28 (7.0)

2016	26	10	5	1	42 (10.6)

2017	35	12	5	3	55 (13.8)

2018	41	13	6	1	61 (15.3)

2019	44	16	6	1	67 (16.8)

2020	62	22	8	4	96 (24.1)

Total	248 (62.3)	95 (23.9)	41 (10.3)	14 (3.5)	398 (100.0)


### Anaesthetic assessment and treatment

Majority (365, 91.7%) were managed by OM, the remaining 33 (8.3%) received NOM. Of those that received OM, 116 (31.8%), 201 (55.1%) and 48 (13.2%) were in ASA III, IV and ASA V respectively. Over four-fifths (28, 84.8%) of cases managed by NOM were injuries from domestic violence, political thuggery, public fights and communal clashes. More than half (208, 57.0%) of the anaesthestic techniques were administered by trainee anaesthetists. Over half (216, 59.2%) of those managed by OM were fixed by intravenous anaesthesia without intubation. About one-third (120, 32.9%) received spinal anaesthesia with or without sedation. The rest (29, 7.9%) were operated under general anaesthesia with endotracheal intubation. Nearly two-third (232, 63.6%) sustained isolated organ/visceral injuries, the remaining 133 (36.4%) sustained multiple intra-abdominal visceral injuries (Table 3a). Head and neck, extremity, chest and maxillo-facial injuries were the most common forms of extra-abdominal injuries (Table 3b). More than one-third (129, 35.3%) had resection with or without stoma while 51 (14.0%) had splenectomy only.

**Table 3a:** Intra-abdominal injury pattern (Isolated and multiple).


ORGAN/VISCERA INVOLVED	FREQUENCY	PERCENT (%)

**Isolated injuries**		

Small intestine	74	20.3

Colon	51	14.0

Spleen	43	11.8

Liver	26	7.1

Retroperitoneal hematoma	14	3.8

Mesentery	11	3.0

Omentum	6	1.6

Stomach	4	1.1

Rectum	3	0.8

Total	232	63.6

**Multiple injuries**		

Small intestine + colon	28	7.7

Spleen + colon + small intestine	20	5.5

Spleen + liver + mesentery	22	6.0

Liver + omentum + mesentery	16	4.4

Colon + retroperitoneal bleed + spleen	14	3.8

Bladder + small intestine	10	2.7

Kidney + colon + spleen	4	1.1

Stomach + diaphragm	4	1.1

Others	15	4.1

Total	133	36.4


**Table 3b:** Distribution of extra-abdominal injuries.


REGION	TYPE OF INJURY	NUMBER OF PATIENTS (N = 78)	PERCENT (%)

Head and Neck (10)	Extradural Hematoma	2	12.8

	Brain Contusion/Concussion	3	

	Cut throat	1	

	Vascular injury	3	

	Cervical injury	1	

Extremity (12)	Open fracture-tibia	1	15.4

	Open fracture-humerus	2	

	Open fracture-radio-ulnar	3	

	Open fracture-tibio-fibular	1	

	Closed fracture-radio-ulnar	1	

	Fracture dislocation	2	

	Digital amputation	2	

Chest (14)	Tension pneumothorax	3	17.9

	Hemothorax +/– rib fracture	4	

	Lung contusion with rib fracture	2	

	Hemopneumothorax	5	

Maxillofacial (8)	Mandibular fracture	2	10.3

	Maxillary avulsion	3	

	Ocular injuries	2	

	Ear avulsion	1	

Urogenital (3)	Penetrating perineal injury	1	3.8

	Urethral injury	1	

	Scrotal hematoma	1	

Integument (12)	Deep avulsion injury	3	15.4

	Deep laceration	6	

	Multiple abrasions	3	

Spine (2)	Lumbar vertebral fracture with neurological deficits	2	2.6

Mixed (17)	Chest/extremity/integument	4	21.8

	Chest/Head and Neck/Maxillofacial	6	

	Integument/extremity	4	

	Chest/Head and Neck	3	

Total		78	100.00


### Outcomes of treatment

The most common complication after operation was wound infection (57, 15.6%). Overall, morbidity and mortality rates in those managed by OM were 29.6% and 12.1% respectively. In the NOM group, mortality occurred in two patients (6.1%). The details of outcomes are shown below (Table 4). Numerous clinical and perioperative parameters impacted on the mortality (Tables 5). Overall, 46 (11.6%) patients died during this study. The main independent predictors of mortality were delayed presentation (P = 0.002), gunshot wounds (P = 0.013), ASA score higher than III (P = 0.001) and bowel resection (P = 0.006).

**Table 4:** Postoperative outcomes.


OUTCOME	MECHANISM OF INJURIES					

	GUNSHOT (%)	STAB(%)	ASSAULT(%)	OTHERS(%)	*χ* ^2^	ODD RATIO

	(N = 244)	(N = 84)	(N = 27)	(N = 10)	(P-VALUE)	(95% CI)

**Morbidity**						

Wound infection	48 (19.7)	6 (7.1)	2 (6.7)	1 (12.5)	3.63 (0.014)	8.32 (5.54–34.66)

Peritoneal abscess	12 (4.9)	2 (2.3)	1 (3.3)	0 (0.0)		

Anastomotic leak	5 (2.0)	1 (1.2)	1 (3.3)	0 (0.0)		

Atelectasis	4 (1.6)	1 (1.2)	0 (0.0)	0 (0.0)		

Sepsis	4 (1.6)	0 (0.0)	1 (3.3)	1 (12.5)		

Incisional hernia	4 (1.6)	1 (1.2)	1 (3.3)	1 (12.5)		

Burst Abdomen	3 (1.2)	0 (0.0)	1 (3.3)	0 (0.0)		

Skin excoriation	3 (1.2)	1 (1.2)	0 (0.0)	0 (0.0)		

Stoma retraction	1 (0.4)	1 (1.2)	1 (3.3)	0 (0.0)		

Total	84 (34.4)	13 (15.5)	8 (29.6)	3 (30.0)		

**LOHs (days)**						

**0–7**	35 (14.4)	16 (19.0)	4 (14.8)	2 (20.0)	8.84 (0.064)	2.16 (0.43–8.44)

8–14	124 (50.8)	42 (50.0)	13 (48.1)	4 (40.0)		

>14	85 (34.8)	26 (31.0)	10 (37.0)	4 (40.0)		

Total	244 (100.0)	84 (100.0)	27 (100.0)	10 (100.0)		

**Mortality**	35 (15.6)	6 (7.1)	2 (7.4)	1 (10.0)	3.86 (0.034)	7.11 (9.12–42.19)


**Table 5:** Effects of clinical and perioperative variables on mortality.


PERIOP PARAMETERS	NO OF CASES	NO OF MORTALITY (%)	*χ*^2^ (P-VALUE)	OR (95% CI of OR)

Mechanism of injury				

Gunshot	248	36 (14.5)	4.73 (0.013)	12.42 (3.52–31.92)

Stab	95	6 (6.3)		

Others	53	4 (7.5)		

Degree of delay (hours)				

0–6	97	6 (6.2)	2.51 (0.002)	7.14 (4.73–33.71)

7–24	112	10 (8.9)		

>24	189	30 (15.9)		

ASA score				

III	116	8 (6.9)	8.24 (0.001)	2.63 (2.15–28.55)

IV	201	23 (11.4)		

V	48	15 (31.3 9		

Method of treatment				

OM	365	44 (12.1)	1.42 (0.024)	11.62 (12.94–44.23)

NOM	33	2 (6.1)		

Status of anesthetist				

Trainee	208	24 (11.5)	13.52 (0.048)	6.32 (4.31–36.11)

Consultant	69	7 (10.1)		

Nurse	88	13 (14.8)		

Age (years)				

16–44	249	27 (10.8)	6.93 (0.033)	3.12 (4.55–22.11)

45–64	117	14 (12.0)		

>64	32	5 (15.6)		

Type of abdominal injury				

Blunt	39	3 (7.7)	2.12 (0.041)	1.21 (7.32–31.11)

Penetrating	359	43 (12.0)		

Intra-abdominal organ involved				

Single	232	23 (9.9)	14.33 (0.034)	9.11 (2.04–19.17)

Multiple	133	21 (15.8)		

Types of operative techniques				

Resection +/– stoma	129	20 (15.5)	3.12 (0.006)	2.11 (4.26–56.22)

Splenectomy only	51	4 (7.8)		

Bowel resect + splenectomy	34	6 (17.6)		

Intestinal repair	32	1 (3.1)		

Proximal diversion	22	1(4.5)		

Evacuation + hemostasis	15	0 (0.0)		

Others	82	12 (14.6)		

Duration of operation (minutes)				

30–60	48	5 (10.4)	3.18 (0.084)	4.16 (0.41–8.14)

61–90	78	8 (10.3)		

91–120	126	16 (12.7)		

>120	113	15 (13.3)		


No = number; OR = odd ratio; CI = confidence interval; ASA = American Society of Aneasthesiologists; OM = operative management; NOM = Non-operative management; resect = resection.

## Discussion

The patients’ population showed male preponderance and comprised mainly young, middle-aged persons who were predominantly farmers, traders and artisans residing in the rural or semi-urban areas. The main mechanism of abdominal injuries was gunshot, perpetrated mostly through armed robbery attacks, communal clashes, political thuggery and cult activities. We observed a steady rise in the rates of abdominal injuries over the years with a disproportionately higher morbidity and mortality rates in those with gunshot wounds. Though majority developed isolated injuries, approximately one third had multiple intra-abdominal visceral trauma. Gunshots, associated injuries (intra-abdominal, head and chest injuries), delayed presentation, advancing age, PAT, high ASA scores, OM and bowel resection and were associated with increased mortality.

In recent time, civilian unrest arising from numerous social, religious and cultural conflicts in Nigeria has reached a crescendo and subsequently given rise to unquantifiable losses of life, disability, social displacement, palpable economic downturn and political insecurity. In the past, Southeast geographical zone of Nigeria enjoyed relative peace with respect to social conflicts, but the ripples of civilian violence from Northern Nigeria spread to the south and became established in the Igbo heartland of Southeast Nigeria. Though the burden of abdominal injuries is a global health challenge, an emerging trend has been recognized in most major cities in sub-Saharan Africa where the emergence of numerous social and political factors that precipitate civilian conflicts and injuries have continued to rise [1,4,5,15,17,18,20,21].

This study showed that 58% of all the abdominal injuries sustained during the period of this study were caused by non-automobile, intentional injuries arising principally from social, religious, political and cultural conflicts. Curiously, published clinical data derived about 30 years ago from a municipal hospital in the old Southeastern Nigeria showed that only 10% of the cases of abdominal injuries resulted from gunshots, stabs and fights [22]. The implication of the findings is that the percentage of abdominal trauma resulting from civilian violence in our setting has leaped approximately six folds in the following three decades or put differently, it has jumped from a tenth to three-fifths of the total cases over the past 30 years. The observed changes in the trend may perhaps, be partly explained by the rising rates of assorted crimes in southern Nigeria and Southeast Nigeria in particular, as cited elsewhere by previous investigators [6,14].

It is noteworthy that the recent upsurge in the rates of abdominal trauma from civilian unrest in our setting may simply mirror the sub-Saharan African-wide epidemiological shift in abdominal injury pattern as other authors from other parts of Nigeria [4,5,6,7,12,18], Ethiopia [1,2,8], Tanzania [20], and Mauritania [17] have documented similar findings. The recent increases in the magnitude of civilian unrest in SSA has been partly ascribed to a rise in access to both local and imported firearms in effort to contain attacks from recurrent wars or sustain agitations by secessionist groups and to perpetrate heinous crimes like terrorism, insurgency, banditry, kidnappings and armed robbery [4,6,12,13,14,20,23,24,25]. It must be emphasized that dwindling economy and security in Nigeria perhaps, paved way for unprecedented crimes executed mostly through social, religious and political conflicts. A recent publication from Southeast Nigeria showed that poor infrastructure, low workforce and deplorable service delivery were rife in the district health centres [26].

Akin to findings in this study, Chianakwanam and colleagues examined a large series of 4,256 patients managed for missiles and blast injuries in a multi-centre study in southeast Nigeria and found that 90.3% and 79.6% were males and aged <50 years [6] respectively. Incidentally, our data and those from the above study [6] overlap with reports from other parts of Nigeria [5,12,13,19,23,25], India [27], Tanzania [15,20], Ethiopia [1], Qatar [28], Mauritania [17], Jordan [29], and Sudan [30]. It has been shown that males and young people are generally more exposed to violence and are more aggressive in demonstrating resistance to perceived threats [9,25]. These observations may partly explain the male and “younger patients” preponderance recorded in this study and other previous studies in SSA [1,5,6,17,20,30], Middle East [28,29], and India [27]. In a Polish study however, the ages of patients ranged between 16–83 years with an average of 41 years and male to female ratio of 1.4:1 [11]. From the foregoing, it appears more females and older patients in European series sustain abdominal injuries compared to their counterparts in SSA [1,2,4,5,16,17,18,20,30], India [27], and Qatar [28]. Elsewhere, it was cited that gunshot injuries in developed countries were mainly due to homicidal and suicidal firearm–related violence [9,11].

In Nigeria, published studies indicate that armed robbery and kidnapping have become very rampant in recent time, mainly for financial rewards [5,9,25,31]. In Umuahia, Southeast Nigeria, Iloh and colleagues found that GSIs were more frequent in middle-aged patients compared to young and elderly persons and ascribed their findings to the fact that middle-aged people are more economically viable and are active working class citizens who have economic wealth that can be stolen by armed robbers or given as ransom to kidnappers [9].

A striking observation was the predominance of GSIs (62.3%) over other mechanisms of injuries, being 2.5 folds, 6 folds, 35 folds and 50 folds higher than stab wounds, assaults, machete wounds and push from heights respectively. In the era of high rates of smuggling and access of firearms into Nigeria and other LMICs, a proportionate rise in crime-related GSIs has become an unfortunate sequela [9,14,25,34]. In this series, armed robbery, kidnappings, communal clashes, political thuggery, cultism and herder-farmers clashes were responsible for 86% of all cases of GSIs consistent with reports from Umuahia [9], Warri [13], and Ado-Ekiti [22], all in southern Nigeria, but varied from reports in Northern Nigeria where insurgency by Islamic militants was responsible for majority of the GSIs [5,11,24].

Surprisingly, most of the abdominal injuries from armed-robbery attacks, kidnappings, communal clashes, cult activities and political thuggery occurred in broad-day light, suggesting a new trend in the operations of crime perpetrators. Report from Umuahia, Nigeria overlap with the above findings [9]. In the past, armed-robbery and cult activities were reserved for night hours to ensure utmost secrecy [9,12,13]. Perhaps, the widespread use of mobile telephones to disseminate and process information speedily, use of cars and modern motorbikes for operations and availability of high-profile riffles and blast devices are responsible for the epidemiological shift in the timing and modus operandi observed in this study.

We observed that in the subset with GSIs, there was a sluggish incremental rate of armed robbery-related GSIs over the eight years of study compared to a steadier rise in the proportions of kidnapping-related GSIs. The above observations indicate that the epidemiology of penetrating abdominal injuries via GSIs in our environment has undergone a remarkable drift. In a referral hospital in southeast Nigeria, Iloh and coworkers observed similar trend and raised a suspicion that the armed robbers may have metamorphosed into kidnappers, possibly as a result of greater financial gains and higher safety profile for the perpetrators of kidnapping compared to armed-robbery attacks that require breaking into houses, shops, banks or other secure places where they are likely to encounter resistance and counter attacks [9]. Investigators from Northeast Nigeria reported comparable results from a survey of insurgency in Northeast Nigeria [5].

In consideration of the foregoing, it can be inferred that inordinate quest for financial gains, political influence, land and natural resources acquisition, fame and assassination plots were the main motives behind gunshot-related activities in this study. The high rates of youths’ unemployment and restiveness, unrestricted access to hard drugs and precipitous decline in moral values among youths may have contributed to the collapse of the moral and social fabrics limiting crime and antisocial behaviors in our setting. Published data from Makurdi [13] and Maiduguri [25], both in Nigeria, Sudan [30], Jordan [29], and Tanzania [15] conform with the above reports.

The fact that majority of the patients sustained PAT highlights the value of injury mechanism in the distribution of injury pattern of the abdomen. Generally, BAT is the predominant pattern of abdominal injuries sustained from RTA, but for injuries sustained through civilian violence, PAT is the usual finding in published series from Nigeria [4,6,18,32,33,34], Ethiopia [1], Sudan [30], and Mauritania [17]. In the classic form, multiple associated intra- and extra-abdominal injuries are frequently found in BAT from RTA while isolated intra-abdominal injuries are commoner with PAT mainly from stab or sharp object wounds, but occasionally from GSIs [4,7,16,17,18,19,32,33]. In a review of 100 consecutive patients with abdominal trauma in Mauritania, Idriss and colleagues found that violent activities from stab injuries were the commonest causes of abdominal trauma and PAT represented 68% of all cases [17]. Majority of the patients sustained isolated intra-abdominal injuries and small bowel, being freely mobile in most of its parts and occupying large spaces in the general peritoneal cavity was the most frequently injured viscera [17]. These findings agree with reports from Gombe, Nigeria [18], Sudan [30], and Ethiopia [1]. In patients with multiple intra-abdominal visceral injuries, we however, observed that GSIs were responsible for majority of cases (Table 3a). It has been found that bullets from high velocity riffles cause tissue destruction, temporary cavitation and elicit spherical shock waves to adjacent structures as it transverses through the tissues [5,6,25]. This mechanism may partly explain the collateral damages produced by GSIs and subsequent involvement of multiple abdominal viscera and extra-abdominal sites consistent with observations made in Maiduguri, Nigeria [5]. From the above discussion, it appears GSIs are versatile in the extent and location of injuries they produce, being capable of wide and extensive/multiple tissue damage when the force or energy of impact is high (or with multiple gunshot wounds), but in the other circumstances, may readily lead to isolated intra-abdominal injuries.

The approach to management of the injured patients in this study was dictated mostly by the type of injury (PAT or BAT), haemodynamic state at presentation, presence or absence of peritonitis, imaging findings and availability of diagnostic facilities to support serial evaluation of patients. The order of frequency of operative treatments done in this study showed that intestinal resection with or without stoma was the most common followed by splenectomy. Published data showed that the wide distribution of intestine and free mobility of the small bowel in the peritoneal cavity make both the small and large bowel vulnerable to traumatic injuries of the abdomen [4,15,16,17,18,32,33,34,35]. Splenectomy or splenic salvage was the second most common procedure in this study. Though the spleen is by far the most commonly affected viscera in BAT compared to PAT, the high rates of multiple visceral injuries involving the spleen and large number of high velocity bullet injuries observed in this study may perhaps, explain the relative high frequency of splenic involvement. Moreover, it has been cited that enlarged, pathological spleens are more vulnerable to traumatic rupture and in Africans where splenomegaly from haemoglobinopathies, chronic pyogenic infection and parasitic infestations are common, it is not surprising that splenic involvement is quite common in abdominal trauma [35,36,37]. We found that majority (84.8%) of cases managed by NOM were sustained through injuries resulting from domestic violence, political thuggery, public fights and communal clashes. The non-penetrating nature of majority of these injuries and the relatively low energy or “impact force” dissipated probably led to lower grades and numbers of visceral injuries and physiologic disturbances. Results from other parts of Nigeria [4,16,18,32,33], Ethiopia [1], and Tanzania [15] support these reports.

In the current discourse, several clinico-pathologic factors impacted on the outcome. In the OM group, long injury-intervention time, advancing age, comorbidity, multiple intra-abdominal and extra-abdominal injuries and intestinal resection were associated with increased mortality. The anatomical extent was largely dependent on the injury mechanism with GSIs accounting for majority of the severe injuries recorded in this study. Similar findings were recorded by researchers in Nigeria [21,38,39] and Tanzania [15]. Though overall perioperative mortality rate (POMR) was 12.1%, specific values for GSIs, stab wounds and others were 14.5%, 6.3% and 7.5% respectively (p = 0.013). These figures emphasize the important contribution of GSIs in accelerating mortality indices in abdominal injury patients. It is noteworthy that GSIs not only accounted for the majority of the morbidity and mortality, it was equally responsible for the severe forms of the morbidities namely deep and organ space surgical site infection (SSI), anastomotic leak, atelectasis, sepsis, burst abdomen and incisional hernias. The most common postoperative complication was SSI (15.6%), the highest incidence being in those with GSIs. Published data by Sheshe and colleagues [4] from Kano and Olaogun and coworkers [32] from Ado-Ekiti, both in Nigeria conform with our results. Our observation that multiple intra-abdominal injuries, delay beyond 12hours, ASA III or more, age > 44years, PAT rather than BAT and bowel resection were associated with increased mortality is comparable with previous published studies [1,15,21].

Delayed presentation was very prominent in this study. Despite the fact that 90.2% of the patients sustained PAT with open wounds, it is disturbing that only 24.4% of the entire patients presented within 6hours to the specialist surgeon. The price for this degree of delay was paid by the higher POMR in those presenting after 24 hours (15.9%) compared to a rate of 6.2% in the early presenters (within 6 hours). In this study, the barriers to early presentation were ascribed to delayed referral by private hospitals or herbalists or patent medicine dealers and failure of patients and relatives to appreciate the clinical magnitude of the abdominal injury and missed injuries due to poor assessment. In a referral hospital in Lagos, Nigeria, Agbroko and colleagues evaluated 76 patients with abdominal injuries and found that the mean injury to intervention time (hours) was 25.4 ± SD 36.4 for the survivors compared to 67.5 ± SD 58.2 for the non-survivors (p = 0.007) [21]. The authors, however attributed the delays to the fact that all their patients were referred by junior doctors who may not appreciate the severity of the injuries and the need for early surgical intervention [21]. Our observations in this study and those from other parts of Nigeria [4,16,18,23,33,34], Tanzania [15], Ethiopia [1], and Sudan [29] tally with the above reports.

## Conclusion

The rate of abdominal trauma from civilian violence is on the steady rise in our setting mostly due to gunshots and stab injuries from armed robbery, kidnapping, communal clashes and political thuggery. PAT affecting mostly the intestine, spleen and liver was the predominant mechanism of injury. Postoperative complications were more common and deadliest in patients who sustained GSIs. Mortality was increased in those with delayed presentation, advanced age >44 years, PAT, multiple intra-abdominal injuries, bowel resection and injuries from gunshots.

## Recommendations

The security apparatuses in southeast Nigeria should be reviewed with a view to formulating a robust security network that can protect lives and properties in the region. In recent time, a government arm of local security network known as “Ebubeagu” was commissioned by the regional government of the five southeastern states of Nigeria. The primary purpose of this outfit is to neutralize the offensive operations of criminal elements that hijacked the activities of a pro-Biafra group known as Indigenous People of Biafra (IPOB). A novel security network referred to as “Eastern Security Network” (ESN) and perceived to be operating under the auspices of IPOB was recently launched to upscale security services in southeast Nigeria, but appears to have long been hijacked by hoodlums with different motives. In response to several massacres by the Fulani herdsmen and incessant kidnappings/banditry in Southeast Nigeria, emergency training with immediate deployment of security personnel known as “forest guards” under the umbrella of “Neighborhood Watch” was commissioned in Southeast Nigeria in 2020. Nevertheless, GSIs and other forms of injuries from civilian violence have continued to rise in Southeast Nigeria despite the presence of the above security networks and the national forces like the police, military and civil defense operating in Southeast Nigeria. In many occasions, ESN operatives have locked horns with the “Ebubeagu” and the national forces.

Recently, abdominal injuries arising from clashes between ESN and Federal government forces, and more importantly, injuries from activities of a clandestine, unidentified militant group known as “unknown gun men” (UGM) swept through the entire Southeast Nigeria leading to steady rise in GSIs and death tolls. Currently, in Southeast Nigeria, Mondays of every week has been forcefully designated “sit-at-home” by IPOB since middle of 2021 till date. Gunshot injuries to the abdomen perpetrated by UGM, ESN, Ebubeagu, government security operatives and criminal elements are witnessed continuously in southeast Nigeria, particularly on Mondays. In view of the foregoing, there is urgent need for the federal and state governments to come up with political and economic blueprints that can restore peace, security and economic growth in the region and other geopolitical zones in Nigeria.

In this study, we found that nearly a tenth (8.1%) of the GSIs were inflicted by police personnel especially by officers under Special Anti-Robbery Squad (SARS), but also through reckless shooting by mobile police personnel (MOPOL), anti-terrorism team (TERROPOL) and other police formations [9,40]. Elsewhere, it has been reported that Nigerian police statutorily assigned to protect the citizens have continued to breed corrupt elements leading to brazen police brutality, extortion of money and valuable possessions like phones and electronic gadgets [9,40]. In Umuahia, Southeast Nigeria, Iloh and colleagues called for change in the professional attitude and practice of Nigerian police towards the use of firearms [9]. In the light of the above, we strongly advocate for a pre-recruitment and serial post-recruitment psychiatric and personality assessments of police and military personnel in Nigeria.

## Limitations

First, the study was carried out in few hospitals in rural southeast Nigeria and our findings may not represent the true status of the epidemiology and outcome of abdominal injuries from civilian conflicts in southeast Nigeria. Many injured patients were ab initio moved to referral and private hospitals and data from such patients were lost. More so, many cases ended up in alternative medicine homes in rural southeast Nigeria and were never admitted to orthodox hospitals. In the light of the above, a more robust prospective study on this subject in southeast Nigeria is warranted.

Second, the unavailability of prehospital emergency medical services and designated trauma centres in our setting is worrisome and hampered the overall outcome measures in this study. Considering the increasing rates of GSIs, and other forms of injuries to the abdomen in our environment in recent time, the establishment of functional prehospital emergency services and designated trauma centres is salutary.

Third, due to presence of other injuries namely head, orthopedic and chest injuries in some of the cases analyzed in this study, the predictive value of abdominal injury as a mortality index in such patients should be interpreted with caution. Fourth, the poor adherence and significant loss to follow up, arguably limited the accuracy of the data presented in this study as some patients were followed up for few months before “drop out”.
